# Comparison of cerebral oxygen desaturation events between children under general anesthesia and chloral hydrate sedation - a randomized controlled trial

**DOI:** 10.1186/s12887-022-03739-8

**Published:** 2022-12-19

**Authors:** Philipp Gude, Thomas P. Weber, Stefan Dazert, Norbert Teig, Philipp Mathmann, Adrian I. Georgevici, Katrin Neumann

**Affiliations:** 1 Department of Anesthesiology and Intensive Care Medicine, St. Josef and St. Elisabeth-Hospital, Ruhr University Bochum, Gudrunstr. 56, D-44791 Bochum, Germany; 2 Department of Otorhinolaryngology, Head and Neck Surgery, St. Elisabeth-Hospital, Ruhr University Bochum, Bochum, Germany; 3 University Children’s Hospital, Ruhr University Bochum, Bochum, Germany; 4 Department of Phoniatrics and Pedaudiology, University Hospital Münster, University of Münster, Münster, Germany; 5 Division of Phoniatrics and Pediatric Audiology, Department of Otorhinolaryngology, Head and Neck Surgery, St. Elisabeth-Hospital, Ruhr University Bochum, Bochum, Germany

**Keywords:** General anesthesia, Child, Chloral hydrate, Near-infrared spectroscopy

## Abstract

**Background:**

During pediatric general anesthesia (GA) and sedation, clinicians aim to maintain physiological parameters within normal ranges. Accordingly, regional cerebral oxygen saturation (rScO_2_) should not drop below preintervention baselines. Our study compared rScO_2_ desaturation events in children undergoing GA or chloral hydrate sedation (CHS).

**Methods:**

Ninety-two children undergoing long auditory assessments were randomly assigned to two study arms: CHS (*n* = 40) and GA (*n* = 52). Data of 81 children (mean age 13.8 months, range 1–36 months) were analyzed.

In the GA group, we followed a predefined 10 N concept (no fear, no pain, normovolemia, normotension, normocardia, normoxemia, normocapnia, normonatremia, normoglycemia, and normothermia). In this group, ENT surgeons performed minor interventions in 29 patients based on intraprocedural microscopic ear examinations.

In the CHS group, recommendations for monitoring and treatment of children undergoing moderate sedation were met. Furthermore, children received a double-barreled nasal oxygen cannula to measure end-tidal carbon dioxide (etCO_2_) and allow oxygen administration. Chloral hydrate was administered in the parent’s presence. Children had no intravenous access which is an advantage of sedation techniques.

In both groups, recommendations for fasting were followed and an experienced anesthesiologist was present during the entire procedure.

Adverse event (AE) was a decline in cerebral oxygenation to below 50% or below 20% from the baseline for ≥1 min. The primary endpoint was the number of children with AE across the study arms. Secondary variables were: fraction of inspired oxygen (F_I_O_2_), oxygen saturation (S_p_O_2_), etCO_2_, systolic and mean blood pressure (BP), and heart rate (HR); these variables were analyzed for their association with drop in rScO_2_ to below baseline (%drop_rScO_2_).

**Results:**

The incidence of AE across groups was not different. The analysis of secondary endpoints showed evidence that %drop_rScO_2_ is more dependent on HR and F_I_O_2_ than on BP and etCO_2_.

**Conclusions:**

This study highlights the strong association between HR and rScO2 in children aged < 3 years, whereas previous studies had primarily discussed the role of BP and etCO_2_. Prompt HR correction may result in shorter periods of cerebral desaturation.

**Trial registration:**

The study was retrospectively registered with the German Clinical Trials Registry (DRKS00024362, 04/02/2021).

## Introduction

Severe neurological complications are rare events after general anesthesia (GA) in early childhood [1]. Possible subclinical effects with consequences for delayed development of cognitive function have also been discussed [2, 3]. In addition, neurological injuries may be caused by intraoperative episodes of low blood pressure (BP), hypocapnia, hyperthermia, hypoglycemia, or hypoxemia [4].

Anesthesia enables necessary surgical or diagnostic procedures to be conducted that are relevant to a child’s development [5]. Diagnostic procedures include, among others, recordings of auditory brainstem responses (ABR) to acoustic stimuli and auditory steady-state responses (ASSR), which are essential methods for the objective determination of a child’s hearing threshold [6–8]. The duration of these measurements often exceeds 1 h because valid assessment of hearing requires broad-spectrum and frequency-specific information and often an additional bone conduction ABR. Therefore, ABR/ASSR of infants aged 3–6 months are frequently recorded under GA or sedation [9]. Thus, we chose to investigate children receiving auditory assessments, based on two arguments: surgical stress plays no role and they are the longest diagnostic procedures in our hospital.

Numerous medical procedures in children are performed under sedation by non-anesthesiologists [10]. Several guidelines provide recommendations for staff and monitoring requirements for different levels of sedation. In Germany, minimally sedated patients require at least pulse oximetry and clinical observation, whereas moderately sedated patients require additional electrocardiography (ECG) and BP monitoring. Capnography is also recommended [11, 12]. Chloral hydrate is still a popular drug for pediatric sedation. Its advantages include easy oral administration, high success rates, and minor adverse effects [13]. The long plasma half-life, sometimes regarded as a disadvantage [12], makes it suitable for lengthy examinations. In general, sedation procedures can be performed very safely in young children. Nonetheless, airway complications, vomiting, agitation, prolonged sleep, and failure to fall asleep have been described [14, 15]. On the other hand, the lack of controlled ventilation and intravenous access, which is actually an advantage of the less invasive procedure, may delay treatment of complications.

The “Safe Anesthesia For Every Tot” initiative (www.safetots.org) defined a criteria-set known as the “10 N concept” that incorporates the following: no fear and accidental awareness, no postoperative discomfort (e.g., no pain), normovolemia, normotension, normocardia, normoxemia, normocapnia, and normonatremia. These criteria aim to maintain homeostasis, thus increasing anesthesia safety in pediatric patients. However, not all physiological reference values are well-defined [16], leading to high variability in clinical practice. For instance, a survey revealed a wide range of BP values considered acceptable by anesthesiologists [17].

Near-infrared spectroscopy (NIRS) enables noninvasive continuous monitoring of regional oxygen saturation of tissues [18]. Previous studies have identified multiple factors influencing regional cerebral oxygen saturation (rScO_2_), including BP, oxygen saturation (S_p_O_2_), heart rate (HR), arterial oxygen content, arterial partial pressure of carbon dioxide, and head and neck positions [18, 19]. Therefore, multiple algorithms have been developed to maintain physiological rScO_2_ and protect the brain by optimizing these factors [19]. Nonetheless, a consensus conference reported insufficient evidence regarding the intraoperative use of NIRS during noncardiac surgery [20]. At this point, it should be noted that superior clinical outcomes from monitoring other physiological values is difficult to prove; for example, using a pulse oximeter has not yet shown improvement in perioperative morbidity and mortality. Nevertheless, it is now considered a standard of care, and most anesthesiologists strive to optimize oxygen saturation [21]. Irrespective of the discussion regarding the possible benefits of NIRS monitoring in terms of outcome, it enables the evaluation of a precisely defined 10 N anesthesia with regard to regional cerebral oxygenation.

This study aimed to compare in children under GA or chloral hydrate sedation (CHS), across study arms, the incidence of adverse events (AE), defined as a drop in rScO_2_ to below 50% or below 20% from the baseline values [19] for at least 1 min.

Our primary hypothesis was that AE incidence is higher in CHS versus GA (χ^2^ test, assumed effect size ≥0.33, power 0.8, alpha 0.05, maximum drop-out 10%, *N* = 75). Secondarily, we measured the association strengths between BP, HR, S_p_O_2_, the fraction of inspired oxygen (F_I_O_2_), end-tidal CO_2_ (etCO_2_), and the dependent variable percentage cerebral oxygenation drop from baseline (%drop_rScO_2_).

## Materials and methods

### Study design and participants

This randomized controlled trial was approved by the Institutional Review Board of the University of Bochum (Reg. No. 16–5720, October 18, 2016) and was conducted according to Good Clinical Practice guidelines and adhered to the principles set forth in the Helsinki Declaration (2013 version). Written informed consent was obtained from the legal representatives of the enrolled children. The study was retrospectively registered with the German Clinical Trials Registry (DRKS00024362). Patients were enrolled in a German university ENT clinic between June 26, 2017, and September 17, 2019.

The attending pediatric audiologist (a physician) recruited children aged up to 3 years, with an American Society of Anesthesiologists physical status I or II, and scheduled for an assessment of ABR/ASSR. Exclusion criteria were body weight < 5 kg, preterm birth, contraindication to anesthesia or sedation, relevant congenital heart disease, sickle cell anemia, acute respiratory infection, and risk of aspiration. Moreover, before study enrollment, all children received tympanometry (226 or 1000 Hz, GSI33TM Tympstar, Grason-Stadler, Minnesota, USA); patients with possible middle ear effusion were excluded.

In clinical practice, parents select for GA or CHS for their children after a detailed consultation. In our study, the detailed counseling was provided by a pediatric audiologist. In addition, we asked the parents for their consent to participate in the study and the necessary randomization. Children whose parents consented to participate in the study were assigned to either the GA or CHS group by a study nurse using coin-flip randomization (although this method is admittedly outdated nowadays, we still consider it valid for the present study, as outlined in the limitations section). The randomization results were documented in a case report file (CRF). Thereafter, a consideration period of at least 24 h was elapsed until the procedures.

### Procedures

Standard monitoring included ECG, oscillometric BP, and S_p_O_2_ (IntelliVue X2 patient monitor, Philips, Boeblingen, Germany). An INVOS™ 5100 cerebral/somatic oximeter (Covidien, Mansfield, USA) with Pediatric Regional Saturation Sensors fixed bilaterally on the forehead was used to measure rScO_2_. That device outputs each second a moving average of the last 5 seconds.

The diagnostic sensitivity of middle ear effusion using tympanometry is mediocre, and despite negative results, suppurative otitis media is expected in up to 30% of cases [7]. Thus, every ABR/ASSR recording under GA was preceded by ear microscopy by an otolaryngologist. Depending on whether suppurative otitis media was suspected, a paracentesis (myringotomy) was performed. This decision had previously been left to the surgeon by the parents through informed consent. This intervention led to the formation of three subgroups: (1) GA for ABR/ASSR only, (2) GA for ABR/ASSR and myringotomy, and (3) CHS.

#### Anesthesia group

The baseline values were recorded before any administration of an anesthetic drug. Premedication (midazolam 0.5–1 mg.kg^− 1^) was given in the parents’ presence in a quiet environment. NIRS probes were attached to the child and baseline values were immediately recorded. In all children, anesthesia was provided by a staff anesthesiologist with expertise in pediatric anesthesia. GA was initiated with sevoflurane in 100% oxygen; after securing an intravenous catheter, boluses of remifentanil 1 μg.kg^− 1^ and propofol 3 mg.kg^− 1^ were administered. GA was maintained with remifentanil (0.3 μg.kg^− 1^.min^− 1^) and propofol (7 mg.kg^− 1^.h^− 1^) as total intravenous anesthesia. The airway was secured with a flexible laryngeal mask airway (LMA®) (Teleflex Medical, Athlone, Ireland). A Draeger anesthetic machine (Primus, Draeger Medical, Luebeck, Germany) was used for ventilation and monitoring of ventilation parameters.

Adherence to all criteria of the 10 N concept was implemented. No fear: midazolam 0.5–1 mg.kg^− 1^ (oral or rectal) up to a maximum of 15 mg was administered to children aged ≥6 months 30 min before entering the operating room. Normotension: the BP measured immediately before GA induction was considered the baseline; hypotension was defined as a decrease in the mean arterial pressure (MAP) to < 38 mmHg or by 20% from the baseline BP, since these values lead to a decrease in cerebral blood flow velocity in infants aged ≤6 months under sevoflurane anesthesia [22]. For safety, this definition was extended to all children. Therapy consisted of a review of the correct depth of anesthesia, an intravenous crystalloid bolus of 20 ml.kg^− 1^ (Jonosteril®, Fresenius, Bad Homburg, Germany), and noradrenalin, if necessary. Normocardia: the threshold for bradycardia was set according to long-term ECG results, with lower limits of 83 beats per minute (bpm) in children aged 0–1 years and 63 bpm in children aged 1–5 years [23]. These children received atropine 10 μg.kg^− 1^, if required. Normoxemia: during induction, the children were pre-oxygenated and ventilated with 100% oxygen; subsequently, F_I_O_2_ was slowly reduced to 0.21. If S_p_O_2_ dropped below 96%, F_I_O_2_ was increased until S_p_O_2_ was 100% again. Normocapnia: the minute volume was adjusted to maintain etCO_2_ in the range of 35–45 mmHg. Normovolemia, normonatremia, and normoglycemia: solid food was allowed for up to 6 h, milk up to 4 h, and clear liquid up to 2 h before anesthesia. Children received a balanced electrolyte solution with 1% glucose (Serumwerk Bernburg, Bernburg, Germany). For the first hour, a 10 ml.kg^− 1^ dosage was chosen to compensate for preoperative fasting. Further dosing followed the 100/50/20 (ml.kg^− 1^.d^− 1^) rule [24]. Glucose levels were determined on an hourly basis after placing intravenous access until emergence from GA. Normothermia: a convective warming system (3 M Bair Hugger®, 3 M Deutschland GmbH, Neuss, Germany) was used, and rectal temperature was measured continuously. No pain: only children undergoing myringotomy received paracetamol or ibuprofen and nalbuphine.

The anesthesiologist-in-charge used standard monitoring. Another anesthesiologist observed the NIRS monitor in a separated part of the operation room.

#### Sedation group

During the pre-procedural consultation, the parents were asked to bring the child in a tired state (e.g., by early waking, keeping them awake in the car). The children received chloral hydrate 50–100 mg.kg^− 1^ orally while resting on either parent’s arm. Chloral hydrate was administered by a pediatric audiologist, in the presence of an anesthesiologist, via a needleless syringe into the child’s buccal pocket so that the child could neither spit out the drug in a large amount nor aspirate it. All children were successfully sedated at the first attempt. As in the anesthesia group, NIRS sensors were attached to the child immediately after the drug administration, when he or she was still awake but cooperative and already calm. Recommendations for monitoring and treatment of children undergoing moderate sedation were met [12]. The etCO_2_ was measured using a double-barreled nasal oxygen cannula (Salter Labs, Arvin, California, USA) that also allows oxygen administration. Children received titrated oxygen if S_p_O_2_ dropped below 96%. They were not cannulated. An anesthesiologist with expertise in pediatric anesthesia was present during the entire examination.

### Statistical methods

The NIRS device logged the rScO_2_ before any drug administration (baseline) and during GA or sedation. The negative percentual deviation was calculated in the data processing: 100 × (rScO_2_-baseline)/rScO_2_, abbreviated as %drop_ rScO_2_, the dependent variable in this study. The BP, HR, S_p_O_2_, F_I_O_2_, and etCO_2_ were considered covariates.

For data processing and statistical analysis, we used R 3.6.2. Distributions of variables were evaluated with the Shapiro–Wilk test. If the distribution was Gaussian, the groups were compared using ANOVA; otherwise, Kruskal–Wallis or Dunn’s test was applied. Sex and AE were analyzed using the χ^2^ test. Our primary hypothesis (H_1_) was that AE incidence would be higher in the sedation group and an assumed effect size ≥0.33; therefore, we expected to reject the null hypothesis H_0_: no statistically significant AE difference across study arms. Using G-Power 3.1.9.2 and χ^2^ goodness-of-fit test, we calculated the sample size as *N* = 75 for *p*-value ≤0.05, power 0.8, drop-out 10%, and non-centrality parameter 7.75.

The secondary endpoint investigated the association strength between covariates and %drop_rScO_2_. For better interpretability of effect sizes, all covariates were centered and scaled by mean and standard deviation, respectively. With this aim, linear regressions were applied in a Bayesian approach, i.e., 1000 bootstrapped models, one sample per child per regression, thus avoiding autocorrelation and overfitting. Lastly, we applied a generalized additive model (GAM) with Gaussian processes tensor product on the predictive attributes to obtain a parsimonious statistical model that considers nonlinear interactions.

## Results

A total of 92 children were initially included in the study and randomized to the CHS (*n* = 40) or GA (*n* = 52) group. The parents of five children did not attend the appointment and three examinations were canceled due to the child’s acute respiratory illness. Three participants were excluded due to interferences of the NIRS device with the ABR signal or the inability to fasten both NIRS and ABR electrodes properly on the forehead.

**Fig. 1:**
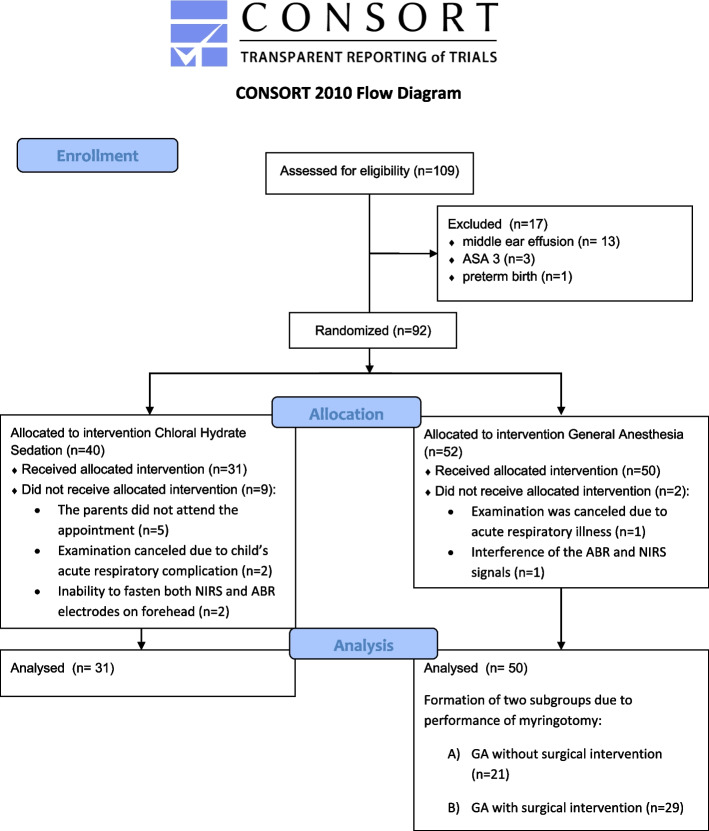
The flow diagram shows the allocation of patients in this randomized controlled trial

Thus, data of 81 children (52 males, 29 females, mean age 13.8 months, range 1–36 months) were analyzed (Fig. 1). Demographic and procedural data are summarized in Table 1, which indicates no demographic between-group differences. Thirty-one patients received sedation and 50 children received GA, 21 of the latter had no surgical intervention. The procedures were shorter in the sedation group than in both anesthesia groups (*p* < 0.01). The median (interquartile range [range]) rScO_2_ baselines were 73 (69.5–79 [57–85]) and 70 (68–74 [62.5–84]) in the anesthesia groups with and without surgery (*p* = 0.33), respectively, and 81 (73–85 [60.5–89.5]) in the sedation group, which was higher than that in the anesthesia groups (both *p* < 0.02).

**Table 1:** Demographic and procedural characteristics of the study cohort

Characteristic	GA with surgery (*n* = 29)	GA without surgery (*n* = 21)	CHS (*n* = 31)
Age (months)	15 (6–23 [3–36])	10 (5–21 [2–34])	8 (4–19.5 [1–31])
Weight (kg)	10 (7.2–12 [5.1–19])	9 (6.8–12 [5–17])	8 (7–10.9 [4.5–16.8])
Sex; female n (%)	10 (34.5)	8 (38.1)	11 (35.5)
Duration of procedures (min)	194.0 (171–204 [127–282])	169.0 (145–206 [55–256])	114.0 (100–143 [70–168])

Values are expressed as median (interquartile range [range]) or numbers and percentages. There were no significant differences in demographic characteristics between the groups (age: GA with surgery vs. GA without surgery *p* = 0.807, GA with surgery vs. CHS *p* = 0.597; GA without surgery vs. CHS *p* = 0.807). The procedures were significant shorter in children undergoing sedation (*p* < 0.01). GA general anesthesia; CHS chloral hydrate sedation

### Comparison of cerebral oxygenation between the groups

The pairwise χ^2^ test across all three groups showed no differences in the incidence of AEs (*p* = 0.95). The statistical test results remained non-significant when the GA groups were merged (*p* = 0.88). Three children, one from each group, met the first criterion of an AE, namely desaturation ≥1 min below 20% from baseline. The child in the sedation group also fulfilled the second criterion for an AE, namely, desaturation < absolute value 50% for 39.7 min. Table 2 summarizes demographic and procedural data of these children. In the anesthesia groups, desaturations below 20% occurred during a decline in HR and were corrected through atropine administration; the child undergoing sedation received supplementary oxygen.

**Table 2:** Children with a drop of regional cerebral oxygen saturation (rScO_2_) ≤50% absolute or ≤ − 20% relative from baseline values for at least one minute

Group	Sex	Age (months)	Weight (kg)	time [rScO_2_ ≤ 50%] (seconds)		time [≤ − -20% from baseline] (seconds)	
				longest single period	total	longest single period	total
Anesthesia with surgery	M	3	6.5	27	27	132	263
Anesthesia without surgery	M	2	5.1	39	39	79	126
Sedation	M	3	6.6	379	2383	352	1417

*M* Male, *F* Female

Although the groups did not differ with respect to the primary endpoint, it is of interest from a practical point of view that in our study, rScO_2_ in children receiving sedation was below baseline for 78% of the procedural time, whereas the anesthesia groups reached significantly lower values: 49% for GA with surgery, and 37% for GA without intervention (sedation vs. GA groups: *p* = 0.01, GA with vs. without surgery: *p* = 0.44).

### Factors influencing cerebral oxygenation

#### Linear models

**Fig. 2:**
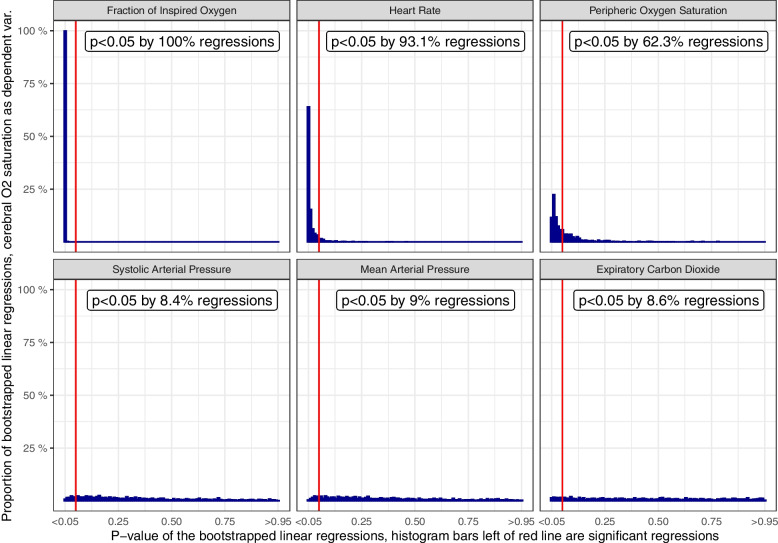
Histogram of 1000 bootstrapped linear regressions between the presented covariates and cerebral oxygen saturation, only the values left of the red line are significant (*p* < 0.05) The values below the variable names indicate the percentage of statistically significant linear regressions, eg., for the fraction of inspired oxygen 1000 out of 1000 bootstrapped regressions (100%) were significant

A total of 1000 bootstrapped linear regressions were fitted on the normalized attributes. The distributions of their statistical significance are displayed in Fig. 2. The BP and etCO_2_ *p*-value distributions were uniform (Fig. 2, second row), indicating no significant effect.

In contrast, the distributions of HR, S_p_O_2_, and F_I_O_2_ were highly left-skewed; their proportions of linear regressions with statistical significance were 100, 93.1, and 62.3%, respectively. Furthermore, the slopes of HR and F_I_O_2_ linear models were HR + 0.456 ± 0.142 (mean ± SD) and F_I_O_2_ + 0.645 ± 0.124, highlighting that both variables have similar high influences on %drop_ rScO_2_.

#### Nonlinear models

**Fig. 3:**
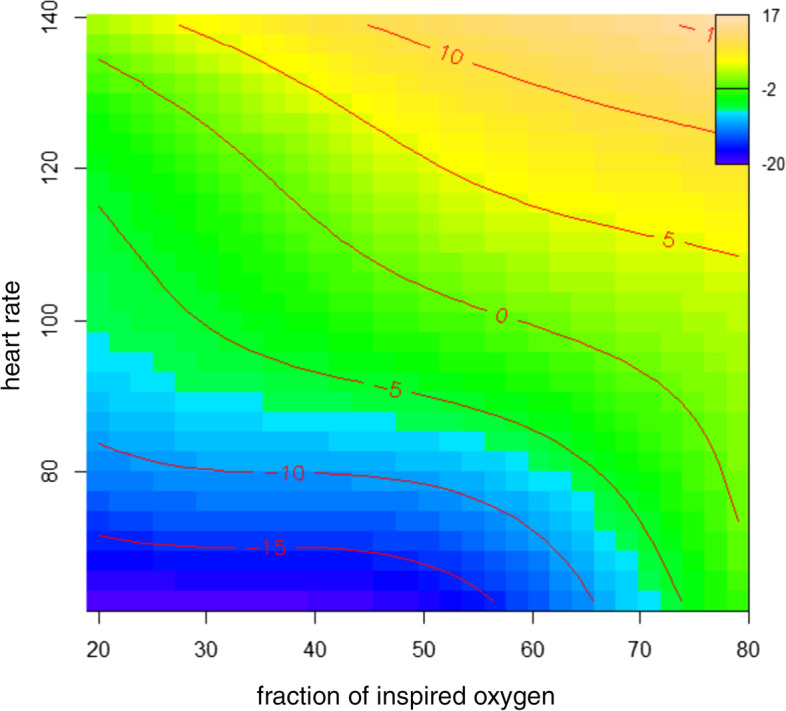
Percentage deviation of rScO_2_ in children receiving anesthesia described with an additive nonlinear model with F_I_O_2_ (horizontal axis) and HR (vertical axis) as independent variables and the percentage deviation of rScO_2_ as a dependent variable. The percentage deviation of rScO_2_ is represented in color and contour lines: the rScO_2_ around baseline are color-coded in green. The intensity of blue represents a negative deviation below the baseline (<− 5%), whereas the yellow color encodes a deviation above the baseline. FiO_2_, fraction of inspired oxygen; rScO_2_, cerebral oxygen saturation

Linear regressions were fitted without interactions; nonetheless, the homeostatic response of the brain to changes in HR and F_I_O_2_ cannot be a priori hypothesized as strictly linear. We extend the statistical modeling by including nonlinear interactions using GAM; this model is presented in Fig. 3. Although including only two covariates (HR and F_I_O_2_), GAM explains 85% of the rScO_2_ variance (*R*^2^ = 0.85). GAM’s highly predictive performance can be explained using tensor products, i.e., interactions (synergy) between covariates. Accordingly, a concomitant increase in HR and F_I_O_2_ should lead to a more effective correction of %drop_rScO_2_.

## Discussion

This randomized controlled trial investigated cerebral oxygenation in children aged up to 36 months undergoing 10 N-based GA or CHS for a long-lasting diagnostic procedure. The primary endpoint compared the incidence of AE across groups (rScO_2_ drop to below 50% or < 20% of baseline for ≥1 min). Hereby, one patient in each group had an AE. The secondary endpoint delivered the current study’s novelty, i.e., the strong influence of HR on cerebral oxygenation, whereas previous works advocated for maintaining BP and etCO_2_ within specific ranges [25, 26].

In this study, 2 of the 50 patients (4%) receiving GA experienced cerebral desaturation. These patients were aged 2 and 3 months, thus having a high risk of critical perioperative events [1]. The proportion of children with %drop_ rScO_2_ below 20% varied in previous studies, possibly due to the different NIRS devices used [27, 28]. In infants aged up to 3 months, including premature infants and patients requiring preoperative mechanical ventilation who underwent complex surgery and GA due to congenital abnormalities or diseases, Razlevice et al. found a %drop_ rScO_2_ below 20% in 18.6% of the children, which is higher than that reported in our study [29]. In a multicenter study, 453 children aged ≥6 months underwent surgery under GA for at least 30 min [30]. Adherence to certain physiological thresholds was not reported. Although their AE definition was more liberal than ours in this study (≥3 min duration), a %drop_ rScO_2_ below 20% from baseline was observed in 6.1% of the patients [30]. Only 2% of the 198 children undergoing noncardiac surgery had a %drop_ rScO_2_ below 20% in a study by Gómez-Pesquera et al. [31]; however, our study cohort was younger.

To date, no human studies have determined the critical rScO_2_ values that represent a hypoxic–ischemic threshold in children [32]. Based on adult data, a decrease in rScO_2_ below 50% absolute or a %drop_rScO_2_ below 20% from baseline appeared to be a reasonable trigger for therapeutic intervention [19, 33]. Interestingly, a recent study revealed a higher incidence of negative postoperative behavioral changes even in children who showed a ≥ 5% decrease in rScO_2_ during noncardiac surgery under GA [31]. Moreover, a recent paper discusses avoiding any rScO2 deviations below awake baselines [34].

In our study, rScO_2_ in the CHS study arm was statistically significant more frequent below the baseline than in the GA groups. One possible interpretation is that closer monitoring and rapid intervention by an anesthesiologist may reduce the duration of suboptimal rScO_2_. In this context, this includes the adherence to the predefined 10 N concept, which can also be seen as a checklist for a structured approach to maintain homeostasis, and has been suggested for children undergoing GA [16]. Notably, full implementation of this concept for sedation techniques without venous access or controlled ventilation, which may be considered less invasive and therefore gentler than GA, is almost impossible. This applies especially to the criteria for normovolemia, normotension, normocardia, normocapnia, and normoglycemia. In the literature, severe AEs from sedation in children have been described [35], especially if they are not carefully planned and safety measures are ignored [36]. In other words, a high process quality is mandatory in any case. For example, at the hospital where this study was conducted, safety measures such as closed-loop communication and the four-eyes principle are integral parts of any anesthesia and sedation procedure. A recently published retrospective study evaluated a sedation protocol in 1331 children undergoing CHS for ABR testing that comprised a structured approach for the overall process, including careful selection of patients, qualification requirements, drug administration, monitoring (HR, S_p_O_2_), and discharge. The procedure resulted in a low AE rate of 0.67%. Of the nine affected children, three developed respiratory complications (two had a mild hypoxia that was successfully treated with head repositioning and one developed evening stridor) [37]. Another large retrospective study on CHS for ABR testing described the clinical course of 697 children. In this study, 3.4% of the children experienced severe AE (apnea, bradycardia) and 6,2% mild AE (including S_p_O_2_ < 90% and vomiting). The authors stated that the attending nurse could resolve all significant AEs, e.g. by repositioning the children’s head and providing supplementary oxygen [38]. Smaller cohorts also showed either no [14] or only transient cardio-respiratory complications that were treatable with simple measures [39, 40]. However, in a review of 1586 children (including 341 aged up to 6 months), 4 patients (0.2%) developed apnea; three of them recovered three patients sponaneously in under 15 seconds, but one child required oxygen ventilation via facemask [41]. In previous randomized controlled trials comparing CHS to intranasal midazolam, dexmedetomidine, or dexmedetomidine plus midazolam in children undergoing ABR testing, no adverse respiratory events were reported in the CHS groups [42–44].

Altogether, these studies demonstrate that a high level of safety can be achieved with sedation procedures, including CHS, but do not allow conclusions to be drawn regarding cerebral oxygenation, because NIRS is not a standard monitoring for sedation in clinical practice or in studies evaluating the safety of a specific sedation protocol. To our knowledge, the present study is the first to analyze rScO_2_ values in children undergoing CHS for a standard diagnostic procedure. Our results encourage the development of quality criteria for monitoring and therapeutic measures that support maintenance of cerebral homeostasis in children during sedation. Padmanabhan et al. studied cerebral oxygenation in 100 children undergoing procedural sedation with various sedatives (ketamine with or without midazolam, fentanyl, pentobarbital, dexmedetomidine, or propofol; no CHS; 33% of the children were premedicated with midazolam). As in our study, severe drops of rScO_2_ values (< 50% absolute or more than 20% from baseline) were rarely observed, but the authors detected a greater effect of hypercarbia on rScO_2_ than hypoxemia [45].

Previous studies revealed that BP is an influencing factor on %drop_rScO_2_ in children undergoing GA [25, 29, 46]. However, defining normotension remains challenging, and few studies on reference values for children aged ≤3 years are available. Determinants are age, sex, size, weight [47, 48], and measurement methods (mercury manometers [47, 49], ultrasound equipment [47], and oscillometry [50, 51]). A wide range of BP values have been described in healthy, non-anesthetized children during their first year of life [51]. Lower reference values were retrospectively determined in anesthetized children [52], which does not indicate that there are generally accepted thresholds for intervention in children of different ages. In our study, the BP targets for the implementation of the 10 N anesthesia were guided by a study that detected a quantifiable association between a decrease in mean arterial pressure (MAP) and cerebral blood flow velocity (CBFV) in infants aged < 6 months [22]. While this is a specific finding in a small population, a dose-dependent decrease in MAP and CBFV has also been demonstrated for propofol [53]. Although the influence of etCO_2_ on rScO_2_ in children under GA has been reported [26], it was not a contributing factor in the anesthesia groups in our study, since the 10 N anesthesia aims at normocapnia.

In our data, F_I_O_2_ and HR showed a greater influence on %drop_ rScO2 than BP and etCO_2_. Michelet et al. examined hemodynamics during intraoperative episodes of cerebral oxygen desaturation in children aged ≤3 months and reported both decreased BP and HR [25]. In contrast, Koch et al. found no correlation between HR and rScO_2_ in neonates undergoing urgent or elective surgery [54].

We had defined normocardia according to previously reported average minimal HR of healthy children during 24-h outpatient ECG recordings [23], expecting these real-life values to be appropriate under GA as well. Following our data analysis, we changed our clinical standard to aim for a higher reference HR as previously described [23]. In addition, our nonlinear model using two variables, HR and F_I_O_2_, accurately predicts %drop_ rScO_2_, providing novel possibilities for maintaining brain homeostasis even when NIRS monitoring is unavailable. Accordingly, a HR of 110–130 bpm and an F_I_O_2_ of 0.3–0.4 would support a stable rScO_2_ for children aged ≤3 years under GA for noncardiac surgery (Fig. 3).

Oxygen delivery (DaO2 = arterial oxygen content × cardiac output) plays a key role in maintaining homeostasis. During anesthesia, F_I_O_2_ and SpO_2_ can be easily controlled by the anesthesiologist. Moreover, in small children, the regulation of cardiac output is primarily dependent on HR. Therefore, our findings fully reflect this fundamental physiological law in the context of pediatric anesthesia. We strongly advocate for maintaining normal (not below baseline) cerebral oxygenation by closely controlling HR and F_I_O_2_.

However, cerebral oxygen delivery also depends on cerebral blood flow (CBF), which is determined by perfusion pressure and cerebrovascular resistance [55], and is also affected by anesthetics. As mentioned above, a dose-dependent decrease in CBFV in healthy children aged 1–6 years undergoing propofol anesthesia has been demonstrated earlier [53]. A remifentanil infusion did not further reduce the CBFV in children anesthetized with propofol, although BP and HR dropped [56]. In our study, these confounding variables were well controlled because of the use of a standardized anesthetic procedure that reflects daily practice. Nonetheless, GA and CHS are quite different interventions that vary in more than one issue. Hence, it remains debatable if direct pharmacological effects, e.g., on cerebral metabolic rate [57] and CBF, might explain the differences in the times below baselines between the groups. In this respect, NIRS values should be considered as endpoints for a variety of possible influencing factors.

Our study has several limitations. First, some children underwent minor surgery based on the results of intraprocedural microscopic ear examinations, which resulted in the creation of a third group. Second, by study design (10 N concept), BP and etCO_2_ were maintained within physiological ranges, i.e. reduced variance; therefore, a precise estimation of their effect-sizes is not feasible. Third, the NIRS sensors were attached to the awake child in the presence of the parents immediately after the administration of chloral hydrate or midazolam. A slight deviation of the baseline compared to a state without external influences cannot completely excluded. Fourth, the groups differed in their adherence to the 10 N criteria in the GA group (not available in the CHS group) and used drugs (GA: midazolam, propofol, remifentanil; sevoflurane, if necessary. CHS: chloral hydrate only). Anesthetic and sedative drugs affect cerebral autoregulation and cerebral metabolism to varying degrees, even within a drug class. These effects can partially depend on the dosage used and carbon dioxide levels [58]. Because all factors influencing cerebral oxygen balance and cerebrovascular reactivity, including CBF, impact rScO_2_ [59], our results may be attributable to other, non-measured parameters. However, the techniques studied here reflect typical procedures in everyday clinical practice. Fifth, the method of coin tossing for randomization of patients to different medical therapies is outdated. Nevertheless, the coin toss used to be an accepted randomization method and was approved by the responsible ethics committee. The flaw attributed to the coin toss is that it can result in a series of identical decisions in succession, which is especially biased in small samples, but this was neither applied nor did it occur in our study.

## Conclusion

The optimized 10 N concept contributes to smaller deviations of cerebral oxygenation from pre-procedural baselines. Our study provides further evidence that HR and F_I_O_2_ are highly correlated with brain oxygenation in children aged up to 3 years old. Our results advocate for the rapid correction of bradycardia to reduce the duration of suboptimal cerebral oxygenation in children below 3 years of age.

## Data Availability

The datasets generated and/or analysed during the current study are not publicly available due permission has not been applied for from neither the participants nor the Ethical Committee but are available from the corresponding author on reasonable request.

## Abbreviations

ABR: Auditory brainstem response
AE: Adverse event
ASSR: Auditory steady-state response
BP: Blood pressure
CHS: Chloral hydrate sedation
ECG: Electrocardiography
etCO_2_: End-tidal carbon dioxide
F_I_O_2_: Fraction of inspired oxygen
GA: General anesthesia
HR: Heart rate
MAP: Mean arterial pressure
NIRS: Near-infrared spectroscopy
rScO_2_: Regional cerebral oxygen saturation
S_p_O_2_: Oxygen saturation
%drop_rScO_2_: Percent cerebral oxygenation drop from baseline
